# A Few Words on the Method of Administering Ether in Surgical Operations

**Published:** 1855-02

**Authors:** 


ART. II.—*A few words on the Method of Administering Ether in Surgical Operations.* By CHARLES MAYOR, M. D., March, 1847. Lausanne: Librairie de George Bridel. 1847.

DR. HUNT:—M. Mayor sent me this paper, (in French,) and as I had no leisure to transcribe it, I handed it to Dr. Lay. You will see that it has been rendered very handsomely; and I send it to you for publication if you think it of sufficient interest. F. H. H.

Extract from the Bulletin of the Société Vaudoise of Natural Sciences.

At the time when the public journals made known the valuable discovery attributed to Messrs. Jackson and Monton, my father was already attacked with the disease which put an end to a life consecrated to humanity and science. I then succeeded him in the duties of Surgeon-in-Chief of the Hospital of Lausanne, a circumstance which furnished me repeated opportunities of proving the effects of the administration of ether, both in the draught and in the state of vapor. Moreover, since the 5th of February, I have been called upon to practice etherization in thirty-seven surgical operations.

Prior to this period, a man suffering from luxation of the humerus, of eight days standing, presented himself at the hospital; but not then having any knowledge of the most proper method of producing inspiration of the ether, I invented an apparatus, which consisted of a bladder, to which was adopted a gum elastic cathetior. Unfortunately the patiennt was so devoid of intelligence as not to be able to understand that he ought to breath through the tube. Explanation and some degree of compulsion proving useless, I reduced the dislocation without the assistance of ether.

Completely foiled in this attempt, I plainly saw that which has also been confirmed by other practitioners, that the success of etherization depends on the choice of the method used in producing it.

The 3d of February, I presented to the Society Vaudoise of Natural Sciences, an apparatus which I had constructed and which is described in its Bulletin. This was the instrument of Charriere simplified, which I have lately seen.

I practiced etherization with this instrument seven times, always with success. I was not slow to believe that instruments of this kind, the only ones that were used at that time, were inconvenient in more ways than one. My father who (although then seriously ill, desirous to assist at two operations)

was struck, as well as myself, with the necessity of an improvement in this respect. In order that etherization may hereafter precede nearly every surgical operation, it is necessary that it be effected in all cases with facility, by the aid of means as simple as possible, and which should not be subject to casualties which could not be surmounted in intractable individuals, in infants, in idiots, lunatics, or animals. It is especially essential that the patient should be able to give vent to the tumultuous loquacity which frequently manifests itself under the influence of ether, and which sometimes degenerates into furious delirium; especially is this the case when the apparatus employed is of such a kind as to cause any obstruction to the facility of utterance. Finally, confinement of the jaws, the outcries which certain persons make in opposition to the operation, the necessity which exists in others of becoming agitated, are so great obstacles as often to render it necessary to discontinue the operation and compromise success.

To arrive at the result which my father and I had in view, we substituted for the tubular apparatus a large and shallow vase such as a shaving dish, a plate, etc., containing some rags and a sufficient dose of ether, (2℥.) This basin was fixed under the chin of the patient in the middle of a moistened towel applied to the head, and the borders of which encircle the vase, the external surface of the inferior maxilla, the occiput and the neck. An assistant maintains the whole in the position indicated, and the patient's face is thus surrounded in an atmosphere loaded with ether, which inhaled through the nose and mouth procure immediate sleep.

This method to which one might have recourse when it was necessary to use some apparatus, is nevertheless not free from inconvenience. In fact the wetted linen conceals the face of the patient from view and permits the escape of a part of the etherial vapor.

I have remedied these disadvantages by substituting for the towel, a glazed veil, which consists of a piece of impermeable cloth,* of 1⅛ yds. in length by twenty-five centimes (nearly a yard) in breadth, and which presents near the centre an open window eighteen centimetres (about 9 in.) high, and fifteen (7 in.) broad. The glass is fixed at an equal distance from the two extremities of the veil in its longest diameter.† Its superior border corres-

---

* I make use of the tissue, the preparation of which I have made known in my memoir on an apparatus for "transuatation et eauvetage," (Bulletin, vol. i. page 298.) It is cotton cloth saturated with linseed oil, dried, and exposed for several days in the shade and in an airy situation.

† In order to fix the glass in the aperture which has been made in the veil, glue all round its edge a little strip of cloth which you can curve to suit the opening.

ponds with a line which divides the veil from end to end, into two equal parts.

The glazed veil is arranged in the same manner as the towel which I before mentioned.* The glass should be placed in front of the patient's face in such a manner that it may be always in view and that nothing which is passing there should escape the notice of the operator.

I have up to this day employed this apparatus in twenty-nine cases of surgical operations, and always with perfect success.

It permits the surgeon to question his patient, and even to hold conversation with him. This instrument, moreover, allows the patient at his choice to breathe through the nose or mouth, to express freely all that he feels, and even to change his position as he has need, without his movements preventing the success of the operation. I have observed, also, that patients more rarely cough and fall asleep sooner when the glazed veil is employed than when we make use of any other method.

Finally, this apparatus is on the whole the most simple, cheapest and most portable. You can, among other forms, give to it that of a cloak or hood with a window, but this modification does not seem to me to present any advantage. It does not belong to my subject to enumerate the divers operations in which I have employed ether. I will content myself by calling the attention of my confreres to a case with which the Gazette Médicalé de Paris has already entertained its readers.† It is in relation to a man 44 years of age, who entered the Hospital of Lausanne, the 5th of February, affected, since the early part of the day, with a strangulated inguinal hernia, against which numerous attempts at reduction had been made. After having prepared every thing for the operation of herniotomy, I submitted the patient to the action of ether, in order to save him the pain of the operation, but with the well-founded hope that the relaxation of the tissue produced by etherization, would enable me to dispense with the use of the knife.

Accordingly, as soon as the patient was rendered insensible, a slight pressure sufficed to effect the return of the intestine.

I will add in closing: That in all cases where I have had recourse to etherization, it has completely produced sleep in the patients, and has rendered them insensible to pain.

That cæteris paribus, the duration of insensibility, depends upon the quan-

---

* Before making use of the apparatus it is well, especially in winter, to rub gently the glass and the vase which is to contain the ether.

† No. of the 20th February, 1847, page 148.

tity of ether which has been respired, and that consequently it is possible, within certain limits, to be prolonged at the will of the operator.*

That the sleep produced by ether, also that the state which precedes it, differ in their nature and duration, since the phenomena which accompany the anæsthesia are due to alcoholic impurities.

That etherization carried so far as to do away with the acute sensibility to pain of the patient, causes no detriment to the health of any one, and does not in the least compromise the success of surgical operations. That finally, as it sometimes produces vomiting, it is well, as far as possible, to avoid administering it to patients while digestion is going on.

---

* I never saw this effect continue so long as to produce any uneasiness. If it should so happen I would not hesitate to administer as an antidote, coffee rather than wine, which has been extolled, and the effects of which are too analogous to those of ether, to be harmless in this case. In the absence of coffee or when this article could not be retained in the stomach, the inspiration of ammoniacal vapor would seem to me to be indicated.

